# The efficacy, safety, and feasibility of inhaled amikacin for the treatment of difficult-to-treat non-tuberculous mycobacterial lung diseases

**DOI:** 10.1186/s12879-017-2665-5

**Published:** 2017-08-09

**Authors:** Kazuma Yagi, Makoto Ishii, Ho Namkoong, Takahiro Asami, Osamu Iketani, Takanori Asakura, Shoji Suzuki, Hiroaki Sugiura, Yoshitake Yamada, Tomoyasu Nishimura, Hiroshi Fujiwara, Yohei Funatsu, Yoshifumi Uwamino, Tetsuro Kamo, Sadatomo Tasaka, Tomoko Betsuyaku, Naoki Hasegawa

**Affiliations:** 1 Division of Pulmonary Medicine, Department of Medicine, Keio University School of Medicine, 35 Shinanomachi Shinjuku-ku, Tokyo, 160-8582 Japan; 2 Department of Pharmacy, Keio University Hospital, Shinjuku-ku, Tokyo, Japan; 3 Department of Diagnostic Radiology, Keio University School of Medicine, Shinjuku-ku, Tokyo, Japan; 4 Keio University Health Center, Minato-ku, Tokyo, Japan; 5 Center for Infectious Diseases and Infection Control, Keio University School of Medicine, Shinjuku-ku, Tokyo, Japan

**Keywords:** Non-tuberculous mycobacterial lung diseases, Inhaled amikacin therapy, Clarithromycin resistance

## Abstract

**Background:**

In multidrug regimens, including an intravenous aminoglycoside (e.g. amikacin [AMK]) is recommended for difficult-to-treat non-tuberculous mycobacterial (NTM) lung diseases. We aimed to evaluate the efficacy, safety, and feasibility of inhaled AMK therapy in patients with difficult-to-treat NTM lung diseases in a retrospective chart review.

**Methods:**

The study population consisted of patients with NTM lung diseases who received combination therapy, including inhaled AMK therapy, at Keio University Hospital (Tokyo, Japan), from January 2014 through May 2016. A total of 26 cases, consisting of 23 *Mycobacterium avium* complex (MAC) and three *Mycobacterium abscessus* complex (MABC) infections cases, were included in this study. The efficacy, safety, and feasibility of inhaled AMK therapy were retrospectively investigated. The Research Ethics Committee of Keio University Hospital approved this study, and informed consent was obtained from all patients.

**Results:**

All 26 patients were culture-positive at enrolment. Twenty-three of the 26 patients (88.5%), including 21/23 MAC patients (91.3%) and 2/3 MABC patients (66.7%), were administered inhaled AMK therapy for >3 months. The proportion of patients who had clinical symptoms, including, cough and sputum, declined after inhalation AMK therapy. Ten of the 23 patients (43.5%) who received AMK inhalation, including 8/21 MAC (38.1%) and 2/2 MABC patients (100%), showed sputum conversion, defined as at least three consecutive negative sputum cultures. Seven of the 23 patients, including, 5/21 MAC and 2/2 MABC patients, showed improvements in high-resolution computed tomography imaging of the chest. In addition, the serum AMK trough levels before the second inhalation were <1.2 μg/mL in all 26 patients, with no occurrence of severe adverse events, such as renal toxicity. One patient (3.8%) experienced auditory toxicity, in the form of tinnitus. However, this symptom was reversible, after temporary interruption of AMK, the patient was able to safely resume the therapy.

**Conclusions:**

Inhaled AMK therapy is an effective and feasible therapy for difficult-to-treat NTM lung disease.

**Electronic supplementary material:**

The online version of this article (doi:10.1186/s12879-017-2665-5) contains supplementary material, which is available to authorized users.

## Background

The increasing prevalence of non-tuberculous mycobacterial (NTM) lung disease is an emerging public health concern worldwide [1–5]. The current treatment statement for NTM lung diseases recommends using long-term multidrug regimens, for example, a regimen of clarithromycin (CLA) or azithromycin (AZM), rifampin (RIP) and ethambutol (EMB) for *Mycobacterium avium* complex (MAC) lung disease [6], but no consensus recommendations have been reached for the treatment of *Mycobacterium abscessus* complex (MABC) lung disease. In addition to the unpredictability of the efficacy of treatment for NTM lung diseases, drug toxicity, drug-drug interactions, intolerance to long-term treatment with multiple antimicrobial agents and resistance to macrolide antibiotics are challenges associated with the management of NTM lung diseases [6–9].

The intravenous administration of aminoglycoside antibiotics, including amikacin (AMK) and streptomycin, is recommended for patients who have rapidly growing mycobacterial or extensive cavitary MAC lung disease, and for those whose treatment with the standard multidrug regimen comprising CLA or AZM, RIP and EMB has failed [6]. However, the systemic administration of aminoglycosides, including AMK, is sometimes associated with major side effects such as auditory toxicity, namely, ototoxicity and vestibular toxicity, and renal toxicity [10]. Therefore, inhaled aminoglycoside therapy achieving high drug concentrations within the lungs could be beneficial for reducing systemic toxicity and drug-drug interactions.

The use of aerosolized antibiotic inhalation is established for the treatment of chronic airway infections caused, for example, by *Pseudomonas aeruginosa* in patients with cystic fibrosis (CF) [11, 12], and for other lower respiratory infections, including non-CF bronchiectasis [13–15]. In addition, inhaled AMK therapy has been used safely and successfully to treat ventilator-associated gram-negative pneumonia in critically ill immunocompetent and immunosuppressed cancer patients [16]. However, few studies have investigated the efficacy and toxicity of inhaled AMK for the treatment of NTM lung diseases [17–19]. We aimed to evaluate the efficacy, safety, and feasibility of inhaled AMK therapy in patients with NTM lung diseases in a chart-based retrospective observational manner.

## Methods

### Study design and study population

The Research Ethics Committee of Keio University Hospital reviewed and approved this study, and written informed consent was obtained from all patients. The study population consisted of patients with NTM lung diseases who received inhaled AMK therapy with combination therapy at Keio University Hospital (Tokyo, Japan) from January 2014 through May 2016. NTM lung diseases were diagnosed using statements published by the American Thoracic Society (ATS) and Infectious Disease Society of America (IDSA) in 2007 [6]. The medical records of 26 patients were retrospectively reviewed, with the data of 23 cases of MAC infection and three cases of MABC infection included in our analysis of the efficacy, safety, and feasibility of inhaled AMK therapy.

### Amikacin inhalation protocol

The inhaled AMK therapy, using injectable AMK sulfate solution (100 mg/mL) without dilution, was initiated at a dose of 15 mg/kg/day once a day for 30 min using a commercially available compressor nebulizer (NE-C28; Omron Colin Co., Ltd., Tokyo, Japan), according to the procedure specified in previous reports [18]. A maximum of 7 mL (700 mg) of injectable AMK sulfate solution was placed in the compressor nebulizer. When the total volume was over 7 mL, nebulization was performed in divided doses. The mode of nebulization was determined based on preliminary detailed data including the nebulizing speeds and particle sizes relating to the compressor nebulizer used in this study (Additional files 1, 2 and 3, Table S1-S3 and Additional files 4, 5, 6 and 7, Figure S1-S4). The duration of the inhaled AMK therapy was determined by the attending physicians based on its efficacy and adverse events; however, at least 3 months of therapy was scheduled.

### Microbiological examinations

The sputum specimens were cultured either in mycobacteria growth indicator tubes (Becton, Dickinson and Company, Sparks, MD, USA) or on egg-based solid media (Kyokuto Pharmaceutical Industrial Co., Ltd. Tokyo, Japan), as previously described [20]. All of the isolates were identified as *Mycobacterium tuberculosis* or NTM using the AccuProbe culture identification test (Gen-Probe Inc., San Diego, CA, USA) and real-time polymerase chain reaction (Cobas Amplicor; Roche Diagnostics, Indianapolis, IN, USA). The NTM species were identified using DNA-DNA hybridization technology (Kyokuto Pharmaceutical Industrial Co., Ltd.). The minimum inhibitory concentration of CLA was determined with BrothMIC NTM using a 7H9 Middlebrook liquid media (Kyokuto Pharmaceutical Industrial Co., Ltd.) formulated using the Clinical and Laboratory Standards Institute’s (CLSI) standard 24A. A minimum inhibitory concentration (MIC) ≥32 μg/mL was defined as CLA resistance [21]. Sputum conversion was defined as at least three consecutive negative sputum cultures after AMK inhalation [22].

### Clinical symptoms

The presence or absence of clinical symptoms, including cough, sputum production, dyspnea, hemoptysis and fever, were evaluated before and at 3 months after the inhaled AMK therapy based on medical records.

### Radiological examination

Chest high-resolution computed tomography (HRCT) scans were evaluated before and three to 6 months after the inhaled AMK therapy by a radiologist and a pulmonologist who were blinded to the patients’ clinical data, and discrepancies between the two were resolved by consensus reviews. The radiological findings following therapy were categorized as “improved”, “unchanged”, or “worsened” [23].

### Adverse events

Patients were monitored throughout inhaled AMK therapy. Renal function was evaluated, based on serum creatinine levels, before and after the inhaled AMK therapy about once every 3 months. Ototoxicity and vestibular toxicity were assessed by an otorhinolaryngologist before the therapy in all cases. After the therapy, assessment by an otorhinolaryngologist was performed when patients complained of symptoms. The serum AMK levels were evaluated 1 h after the first AMK inhalation began, and immediately before the second inhalation.

### Statistical analysis

The summary statistics for the quantitative markers are presented as the medians and interquartile range. Differences were analyzed by McNemar’s non-parametric test for paired proportions.

## Results

### Patients’ characteristics

Twenty-six patients who met the ATS/IDSA statement’s diagnostic criteria for NTM lung diseases [6] received inhaled AMK. All of patients had negative test results for the human immunodeficiency virus. Table 1 presents patients’ characteristics. The majority of patients (84.6%) were women, and the median age of the cohort at the time of initiating AMK inhalation was 65.5 years (interquartile range, 60.0-70.5 years). Three patients (11.5%) (Case numbers 1, 3 and 7) were former smokers. All 26 patients had a positive culture at the beginning of AMK inhalation, with 23 patients (88.5%) having a MAC infection and three patients (11.5%) a MABC infection. The median time from diagnosis to the start of treatment was 73.5 months (interquartile range, 38.5-131.0 months). *Pseudomonas aeruginosa* (*n* = 3, 11.5%) and *Aspergillus* spp. (*n* = 3, 11.5%) were the most common concomitantly isolated organisms when therapy was initiated.

**Table 1:** Patient background data (*n* = 26)

Backgrounds median [interquartile range] or number (%)	
Age, years	65.5 [60.0-70.5]
Male/Female	4 (15.4) /22 (84.6)
Weight, kg	44.0 [41.7-47.7]
BMI, kg/m^2^	18.0 [17.3-19.4]
Smoking history	3 (11.5)
Mycobacterium species	
*Mycobacterium avium* complex	23 (88.5)
*M.avium*, *M.intracellulare*	22 (84.6), 1 (3.8)
*Mycobacterium abscessus* complex	3 (11.5)
Medical history	
Chronic obstructive pulmonary disease	1 (3.8)
Bronchial asthma	1 (3.8)
Interstitial pneumonia	2 (7.7)
Bacterial pneumonia	2 (7.7)
Old pulmonary tuberculosis	1 (3.8)
Empyema	1 (3.8)
Pulmonary aspergillosis	1 (3.8)
Pneumothorax	2 (7.7)
Rheumatoid arthritis	4 (15.4)
Chronic sinusitis	3 (11.5)
Time from diagnosis until the initiation of treatment, months	73.5 [38.5-131.0]
Concomitant organisms at the initiation of inhaled AMK therapy	
*Pseudomonas aeruginosa*	3 (11.5)
*Aspergillus* species	3 (11.5)

### Case data summaries

Reasons for initiation of AMK therapy included severe progressive disease, adverse drug reactions to other agents, possible drug-drug interactions, and the presence of CLA-resistant MAC lung disease. In 11 of the 23 MAC patients, the therapy was initiated for severe progressive diseases In three patients (Case numbers 1, 5, and 13), the therapy was initiated for adverse drug reactions due to other agents and possible drug-drug interactions. In nine patients (Case numbers 2, 7, 9, 12, 14, 15, 18, 23, and 24), the therapy was initiated owing to the presence of CLA-resistance.

Table 2 summarizes the case data for the 26 patients with NTM lung diseases who received inhaled AMK therapy. Nine of the 23 MAC patients (39.1%) had CLA-resistant isolates (Table 2). The distribution of NTM types on HRCT images among MAC patients was as follows: nodular bronchiectatic (NB) type, 10/23 (43.5%); fibrocavitary (FC) type, 1/23 (4.3%); and NB + FC type, 11/23 (47.8%). Among the entire cohort, 13/26 (50.0%) patients (11 MAC, 2 MABC) had cavitary lesions. The median duration of AMK therapy was 61.0 months (interquartile range, 29.5-104.3 months).

**Table 2:** Clinical characteristics of patients with nontuberculous mycobacterial lung disease receiving inhaled amikacin therapy

Case	Sex	Species	MIC of CLA (μg/mL)	Radiological pattern	Prior Treatment	Total treatment duration before AMK therapy (months)	Drugs at AMK initiation	Duration of AMK therapy (months)	Sputum conversion post-therapy	Radiological findings poet-therapy
#1	M	*M.avium*	0.25	FC	CLA, EMB	16	CLA, EMB	13	+	Improvement
#2	F	*M.avium*	0.5	NB + FC	CLA, RIP, EMB	4	CLA, RIP, EMB	7	−	No change
#3	M	*M.avium*	>32	NB	CLA, RIP, EMB	52	RIP, EMB, STFX	6	−	Worsening
#4	F	*M.avium*	0.5	NB	CLA, RIP, EMB	124	CLA, RIP, EMB	5	+	No change
#5	F	*M.intracellulare*	≦0.03	NB + FC	CLA, EMB	9	CLA, EMB	20	−	Worsening
#6	M	*M.avium*	0.5	NB	CLA, RIP, STFX, AMK div	110	CLA, RIP, STFX	1^a^	Unevaluable	Unevaluable
#7	F	*M.avium*	>32	NB + FC	RIP, STFX	16	RIP, STFX	< 1^a^	Unevaluable	Unevaluable
#8	F	*M.avium*	2	NB	CLA, RIP, EMB	43	CLA, RIP, EMB	36^b^	−	No change
#9	F	*M.avium*	>32	NB	RFB, MFLX	221	RFB, MFLX	6	−	Worsening
#10	F	*M.avium*	0.5	NB	CLA, RIP, EMB, STFX	86	CLA, RIP, EMB, STFX	6, 7^c^	−	No change
#11	F	*M.abscessus*	−	NB	EMB, STFX	29	EMB, STFX	24	+	Improvement
#12	F	*M.avium*	>32	NB + FC	CLA, EMB	99	CLA, EMB	7, 9^c^	+	Worsening
#13	F	*M.avium*	0.5	NB	CLA, RIP, STFX	72	CLA, RIP, STFX	6	−	No change
#14	F	*M.avium*	>32	NB + FC	RIP, EMB, STFX	145	RIP, EMB, STFX	6	+	Unchanged
#15	F	*M.avium*	>32	NB	RIP, EMB, STFX	81	RIP, EMB, STFX	13	+	Improvement
#16	F	*M.avium*	4	NB + FC	CLA, RIP, EMB	67	CLA, RIP, EMB	24	+	Improvement
#17	F	*M.abscessus*	−	NB + FC	CLA, STFX, DOXY	31	STFX, DOXY, DRPM	3^a^	Unevaluable	Unevaluable
#18	F	*M.avium*	>32	NB	EMB, STFX	55	EMB, STFX	6, <1^c^	−	No change
#19	F	*M.avium*	4	NB + FC	CLA, RIP, EMB	112	CLA, RIP, EMB	7	−	No change
#20	F	*M.avium*	0.125	NB + FC	CLA, RIP, EMB	13	CLA, RIP, EMB	12	+	Improvement
#21	F	*M.abscessus*	−	NB + FC	CLA, RIP, EMB	25	CLA, DOXY, DRPM	12	+	Improvement
#22	M	*M.avium*	0.125	NB	CLA, RIP, EMB, STFX	83	CLA, RIP, EMB, STFX	10	−	Worsening
#23	F	*M.avium*	>32	NB	CLA, RIP, EMB	131	RIP, EMB	6	−	No change
#24	F	*M.avium*	>32	NB + FC	RIP, EMB, STFX	106	RIP, EMB, STFX	7, 6^c^	−	No change
#25	F	*M.avium*	0.25	NB	CLA, RIP, EMB	46	CLA, RIP, EMB	9	+	Improvement
#26	F	*M.avium*	0.125	NB + FC	CLA, RIP, EMB	44	CLA, RIP, EMB	6	−	No change

*M* male, *F* female, *div* intravenous drip

*CLA* clarithromycin, *EMB* ethambutol, *RIP* rifampicin, *RFB* rifabutin, *STFX* sitafloxacin, *DRPM* doripenem, *DOXY* doxycycline, *NB* nodular bronchiectatic type, *FC* fibrocavitary type

(under line); CLA resistance (CLA MIC ≥32 (μg/mL))

^**a**^loss to follow-up

^**b**^current therapy is also being continued

^**c**^inhaled AMK therapy was used again at some interval

Sputum conversion ‘+’ denotes three consecutive negative sputum cultures during follow up

AMK therapy was discontinued within 1 month after initiation in two patients, one (case 6) due to dysphonia, which completely recovered within 10 days of AMK therapy cessation, and the other (case 7) due to the development of a pneumothorax, which was likely an AMK-unrelated adverse event). One patient (case 17) was transferred to another hospital 3 months after treatment initiation and was lost to follow-up.

**Fig. 1:**
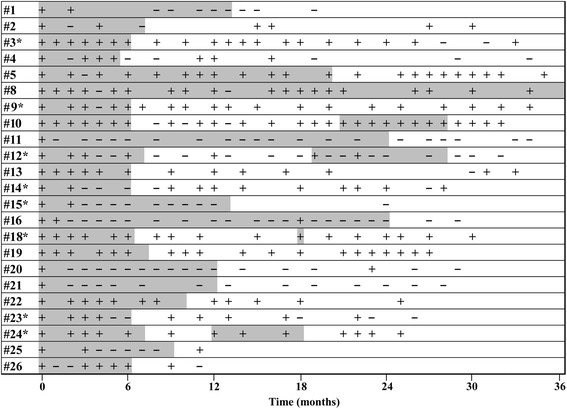
Sputum culture results over time of each case. Halftone pattern indicates the period during inhaled AMK therapy. * indicates CLA-resistance (CLA MIC ≥32 [μg/mL]). “+” denotes positive sputum culture. “-” denotes negative sputum culture

Twenty-three patients with NTM lung diseases (88.5%), including 21/23 MAC patients (91.3%) and 2/3 MABC patients (66.7%), were administered inhaled AMK for >3 months. Ten of these patients (43.5%), including 8 of the 21 MAC patients (38.1%) and the 2 MABC patients (100%) showed sputum conversion (Table 2, Fig. 1).

AMK therapy was also administered for >3 months in 8/9 MAC patients and CLA-resistance, with sputum conversion achieved in 3 cases (12, 14 and 15; 37.5%). Only one patient (case 14) showed microbiological recurrence, defined as more than two consecutive positive sputum culture results after sputum conversion (Table 2, Fig. 1).

In four patients, (cases 10, 12, 18, and 24), inhaled AMK therapy was reinitiated at an interval for various reasons. Therapy was reinitiated in case 10 at 14 months after termination of the initial course of inhaled AMK therapy due to worsening respiratory symptoms, including hemoptysis and worsening of HRCT findings. Case 12 showed sputum conversion with inhaled AMK therapy, in spite of having CLA-resistant MAC lung disease. However, AMK therapy was reinitiated at 11 months after termination of the initial course of therapy, in combination with CLA and EMB, to prevent the progression of MAC disease with the initiation of methotrexate therapy for progressing rheumatoid arthritis (RA). Similar to case 10, AMK therapy was reinitiated in case 18 due to worsening respiratory symptoms, including coughing and HRCT findings, at 12 months after the initial course of therapy. However, inhaled AMK therapy was discontinued 2 weeks after re-initiation, upon the patient’s request, due to mild throat discomfort that resolved quickly after discontinuation. Case 24 received inhaled AMK therapy again 4 months after completion of the initial treatment due to worsening respiratory symptoms, including coughing and sustained positive sputum culture.

Among the patients receiving inhaled AMK for >3 months, 5/21 MAC patients (23.8%) and 2/2 MABC patients (100%) exhibited improvements in their HRCT findings. The HRCT findings were unchanged for 11/21 MAC patients (52.4%) and worsened in 5/21 MAC patients (23.8%) during treatment. Among patients showing sputum conversion, 5/8 MAC patients and 2/2 MABC patients exhibited improvements on their HRCT findings (Table 2).

### Clinical symptoms before and after inhaled amikacin therapy

Table 3 presents the percentage of patients who had clinical symptoms, including coughing, sputum, dyspnea, hemoptysis, and fever, before and after treatment. The proportion of patients who demonstrated coughing and sputum significantly decreased after AMK treatment.

**Table 3:** Clinical symptoms before and after amikacin treatment (Number [%])

Clinical symptom	Before treatment (*n* = 23)	After treatment (*n* = 23)	*P* value^a^
Cough	22 (95.7)	16 (69.6)	0.0412
Sputum	22 (95.7)	12 (52.2)	0.0044
Dyspnea	15 (65.2)	14 (60.9)	>0.99
Hemoptysis	5 (21.7)	3 (13.0)	0.4795
Fever	1 (4.3)	0	>0.99

^a^McNemar’s test

### Adverse events after inhaled amikacin therapy

There were no severe systemic adverse events, such as renal toxicity although 13 of the 26 patients (50.0%) developed some type of toxicity due to inhaled AMK therapy; one patient (3.9%) was diagnosed with auditory toxicity of tinnitus after the commencing AMK inhalation therapy for 1 month, which lead to the interruption of therapy for 2 weeks. However, this symptom was tolerable, and the patient’s condition improved sufficiently to resume therapy. Twelve patients (46.2%) had uncomfortable sensations in their oral cavities, one patient (3.8%) had oral candidiasis, while five patients (19.2%) experienced hoarseness. However, these adverse events were tolerable and the AMK inhalation therapy was continued (Table 4).

**Table 4:** Adverse event profiling of amikacin inhalation (*n* = 26)

Adverse event	Number (%)
Auditory toxicity	1 (3.8)
Renal toxicity	0 (0.0)
Uncomfortable feeling in the oral cavity	12 (46.2)
Oral candidiasis	1 (3.8)
Hoarseness	5 (19.2)
Dysphonia	1 (3.8)
Pneumothorax	1 (3.8)
Hemoptysis	1 (3.8)
Digestive symptom	1 (3.8)
Vertigo	1 (3.8)
Epistaxis	1 (3.8)

### Serum amikacin concentrations

The serum AMK trough concentrations, measured immediately before initiation of the second AMK inhalation, were <1.2 μg/mL in all 26 patients (Table 5). Most patients (23 out of 26) showed low serum AMK levels of ≤2.4 μg/mL 1 h after the AMK therapy, which is regarded as the peak serum concentration, this level being much lower than the serum AMK level following intravenous therapy [10].

**Table 5:** Serum amikacin concentrations (*n* = 26)

Serum amikacin concentrations (μg/mL)	n (%)
(A) Serum amikacin concentrations just before the start of amikacin inhalation (trough concentrations)	
< 0.8	24 (92.3)
0.8 to <1.2	2 (7.7)
≥ 1.2	0 (0)
(B) Serum amikacin concentrations 1 h after the start of amikacin inhalation	
< 0.8	4 (15.4)
0.8 to <1.2	6 (23.1)
1.2 to <1.6	8 (30.8)
1.6 to <2.0	3 (11.5)
2.0 to <2.4	2 (7.7)
≥ 2.4	3 (11.5)

## Discussion

Through a retrospective evaluation of 26 patients with NTM lung diseases, we demonstrated that the addition of inhaled AMK therapy was safe and effective for patients with difficult-to-treat NTM lung diseases. The therapy could be administered therapy for >3 months in most patients (88.5%) without severe adverse events. Among 23 patients who successfully inhaled AMK once a day for >3 months, 15 patients (65.2%) showed at least one instance of a negative sputum culture, with 10 patients (43.5%) showing sputum conversion after treatment. Moreover, eight patients with CLA-resistant MAC were administered inhaled AMK therapy for >3 months, with three patients (37.5%) achieving sputum conversion post treatment. Furthermore, HRCT findings improved in seven of the 23 MAC patients, with concurrent improvement of clinical symptoms.

Three reports have described inhaled AMK therapy for NTM lung disease [17–19]. A recent report showed that 40% of the patients with NTM lung diseases who were administered inhaled AMK therapy had at least one negative sputum culture [17], which is lower than that observed in this study (65.2%). This could be attributed to previous studies using lower AMK doses than we did in our study (>500 mg/day; median dose, 600 mg; interquartile range, 600–700 mg), or the inclusion of more severe cases, as evidenced by the finding that the proportion of CLA-resistant NTM lung diseases (75%) was more than that of the current study (39.1%). An observational case series study of six patients with MAC lung diseases demonstrated a higher response rate (83.3%) to AMK inhalation in relation to sputum conversion and symptoms [18] compared with the current study, which was possibly due to longer therapy duration (median, 24.5; range, 4–52 months) than current study (median, 7.0; interquartile range, 6.0–12.5 months). The third AMK inhalation study also showed a high response rate (88%) that was based on symptom improvements [19], which concurs with our results that showed improved clinical symptoms (Table 3). Collectively, these results indicate that inhaled AMK therapy would be effective in selected patients with intractable NTM lung diseases.

We monitored the serum AMK concentrations following AMK inhalation. Our data suggested that a limited amount of inhaled AMK entered the bloodstream through the airway’s epithelial surface, which in turn, meant a large part of the inhaled AMK was compartmentalized within the lung. No severe adverse events, including renal toxicity, occurred. Auditory toxicity of tinnitus occurred in only one patient, but this symptom was tolerable and the patient improved to resume the therapy. One patient stopped AMK inhalation because of dysphonia, probably due to inhaled AMK, but completely recovered in 10 days after ceasing inhalation. Hence based on the present analysis, inhaled AMK would be a safe and feasible therapy.

While the ATS/IDSA statement [6] recommends that intravenous AMK therapy be of relatively short duration (2–3 months), possibly because of adverse events, firm recommendations do not exist regarding AMK inhalation therapy. Referring to previous studies, therefore we tried to administer AMK inhalation for >3 months (median, 7.0; interquartile range, 6.0–12.5 months). While the number is limited, given the safety and effectiveness of inhaled AMK, relatively longer durations, that is, >2-3 months, of inhaled AMK therapy in combination with multiple drug chemotherapy could be promising for difficult-to-treat NTM lung diseases. Indeed, the median durations of inhaled AMK therapy in the previous studies were 19 months (range, 1–50) [17], 24.5 months (range, 4–52) [18] and 75 days (range, 18–277) [19].

Since it is obvious that nebulizing conditions are critically important for directly influencing the efficacy of inhalation results, we collected fundamental information in advance about the compressor nebulizer used in this study, from then on we chose domestic compressor nebulizers for this study. Five different conditions were evaluated by modulating the nebulizing times and speeds, and we measured the final remaining doses and the particle sizes under each condition to choose the most suitable mode for our cases (Additional file 1, Table S1).

Recently, liposomal amikacin for inhalation (LAI) has been developed [22]. A phase 2 study, investigating the efficacy and safety of 590 mg/day of LAI for pulmonary nontuberculous mycobacterial disease that was difficult-to-treat with ordinary chemotherapy, was published [22]. A greater proportion of the LAI group demonstrated ≥1 negative sputum cultures (32% [14/44] vs. 9% [4/45]; *p* = 0.006) [22], which is lower than that observed in this study (65.2%) and the sputum culture conversion rate (43.5% [10/23]). Systemic adverse events observed in this study such as auditory toxicity (3.8%) and renal toxicity (0.0%) were lower than that observed in the report using LAI (11.4% and 2.3%, respectively). It is important to note that the study designs were different; specifically, as the LAI study was a formal prospective clinical trial, it is likely to detect more side effects. The LAI trial also included patients with CF, for whom treatment is more difficult than for NTM lung disease. A phase 3 clinical trial is in progress and is evaluating the efficacy of LAI when added to a multi-drug regimen compared to a multi-drug regimen alone (NCT02344004).

This study has several limitations. First, the current study is a retrospective analysis using limited number of cases, which is not appropriate to affirm the safety and feasibility of inhaled AMK. Second, the duration of observation did not enable evaluation of the long-term effectiveness and toxicity of AMK inhalation therapy. Third, sputum culture was not strictly performed at each visit to clinic to assess microbiological conversion. While this is a retrospective analysis without a designated protocol, sputum samples should have been more frequently obtained using appropriate sputum induction. Fourth, there is a possibility that sputum culture conversion may be due to the presence of AMK, although we collected sputum samples before administrating the inhaled AMK therapy, to avoid (or minimize) the effect of AMK. Finally, it was difficult to determine the exact beneficial effect of inhaled AMK therapy only in the current study, because we used AMK inhalation to treat NTM infections in patients with a variety of underlying lung diseases in combination with systemic chemotherapy in some cases. Therefore, treatment responses may have occurred due to the combined systemic chemotherapy, rather than inhaled AMK therapy alone. These issues may be resolved by undertaking studies that involve the administration of AMK inhalation therapy as an add-on for patients who are difficult-to-treat with the guideline-based standard treatment and that have sufficiently long observation periods.

## Conclusions

Inhaled AMK therapy is effective, safe and feasible for intractable NTM lung diseases. Randomized controlled studies involving large numbers of patients and longer follow-up periods are required to evaluate the benefits and risks associated with inhaled AMK therapy.

## Additional files

Additional file 1: Table S1. Five different conditions were tried for the nebulization of amikacin sulphate. (DOCX 24 kb)

Additional file 2: Table S2. Nebulizing time and speed, final remaining dose and the particle size in the compressor nebulizer. A digital weighing scale was used to measure the mean nebulizing speed and the final remaining dose. The particle sizes were measured using a Mastersizer 2000 (Malvern Instruments Ltd., Worcestershire, UK). (DOCX 186 kb)

Additional file 3: Table S3. Time course changes in the total weights, remaining doses and nebulizing speeds. The underlined numbers indicate the weight data measured immediately after the amikacin solution was added. (DOCX 677 kb)

Additional file 4: Figure S1. The equipment used to measure the aerosolized particle sizes (Mastersizer 2000: Malvern Instruments Ltd., Worcestershire, UK). (DOCX 251 kb)

Additional file 5: Figure S2. Particle sizes and solution volumes in condition 1, condition 2 and condition 5. The aerosolized particle sizes were measured using a Mastersizer 2000 (Malvern Instruments Ltd., Worcestershire, UK). (DOCX 243 kb)

Additional file 6: Figure S3. Time course changes of the remaining doses. (DOCX 282 kb)

Additional file 7: Figure S4. Time course changes of the nebulizing speeds. (DOCX 312 kb)

## Abbreviations

AMK: Amikacin
CLA: Clarithromycin
DOXY: Doxycycline
DRPM: Doripenem
EMB: Ethambutol
FC: Fibrocavitary type
MABC: *Mycobacterium abscessus* complex
MAC: *Mycobacterium avium* complex
MIC: Minimum inhibitory concentration
NB: Nodular bronchiectatic type
NTM: Non-tuberculous mycobacterial
RFB: Rifabutin
RIP: Rifampicin
STFX: Sitafloxacin
