# Supplementary material for: The efficacy, safety, and feasibility of inhaled amikacin for the treatment of difficult-to-treat non-tuberculous mycobacterial lung diseases
Source: BMC Infect Dis. 2017 Aug 9;17:558. doi: 10.1186/s12879-017-2665-5 (PMC5550988; doi:10.1186/s12879-017-2665-5)
Supplement: Supplementary file 1 — Five different conditions were tried for the nebulization of amikacin sulphate. (DOCX 24 kb) [file 12879_2017_2665_MOESM1_ESM.docx]

**Table S1** Five different conditions were tried for the nebulization of amikacin sulphate.

| **Condition 1** | Undiluted 7 mL solution of amikacin sulphate (100 mg/mL) |
| --- | --- |
| **Condition 2** | Neat 3.5 mL solution of amikacin sulphate (100 mg/mL) diluted with 1 mL saline to a total volume of 4.5 mL, repeated twice (9 mL volume in total) |
| **Condition 3** | Undiluted 4.5 mL solution of amikacin sulphate (100 mg/mL), repeated twice (9 mL volume in total) |
| **Condition 4** | Undiluted 5.5 mL solution of amikacin sulphate (100 mg/mL), repeated twice (11 mL volume in total) |
| **Condition 5** | Neat 3.5 mL solution of amikacin sulphate (100 mg/mL) diluted with 2 mL saline to a total volume of 5.5 mL, repeated twice (11 mL volume in total) |
