# Supplementary material for: The efficacy, safety, and feasibility of inhaled amikacin for the treatment of difficult-to-treat non-tuberculous mycobacterial lung diseases
Source: BMC Infect Dis. 2017 Aug 9;17:558. doi: 10.1186/s12879-017-2665-5 (PMC5550988; doi:10.1186/s12879-017-2665-5)

**Table S2** Nebulizing time and speed, final remaining dose and the particle size in the compressor nebulizer. A digital weighing scale was used to measure the mean nebulizing speed and the final remaining dose. The particle sizes were measured using a Mastersizer 2000 (Malvern Instruments Ltd., Worcestershire, UK).


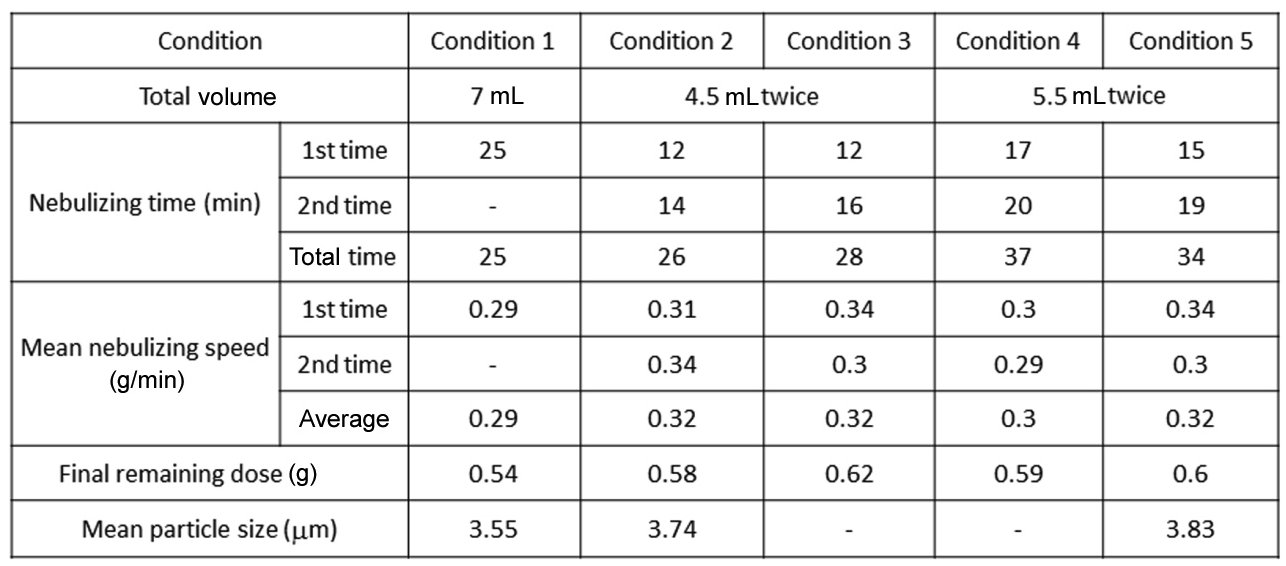

Supplement: Supplementary file 2 — Nebulizing time and speed, final remaining dose and the particle size in the compressor nebulizer. A digital weighing scale was used to measure the mean nebulizing speed and the final remaining dose. The particle sizes were measured using a Mastersizer 2000 (Malvern Instruments Ltd., Worcestershire, UK). (DOCX 186 kb) [file 12879_2017_2665_MOESM2_ESM.docx]
