# Supplementary material for: The efficacy, safety, and feasibility of inhaled amikacin for the treatment of difficult-to-treat non-tuberculous mycobacterial lung diseases
Source: BMC Infect Dis. 2017 Aug 9;17:558. doi: 10.1186/s12879-017-2665-5 (PMC5550988; doi:10.1186/s12879-017-2665-5)

**Table S3** Time course changes in the total weights, remaining doses and nebulizing speeds. The underlined numbers indicate the weight data measured immediately after the amikacin solution was added.


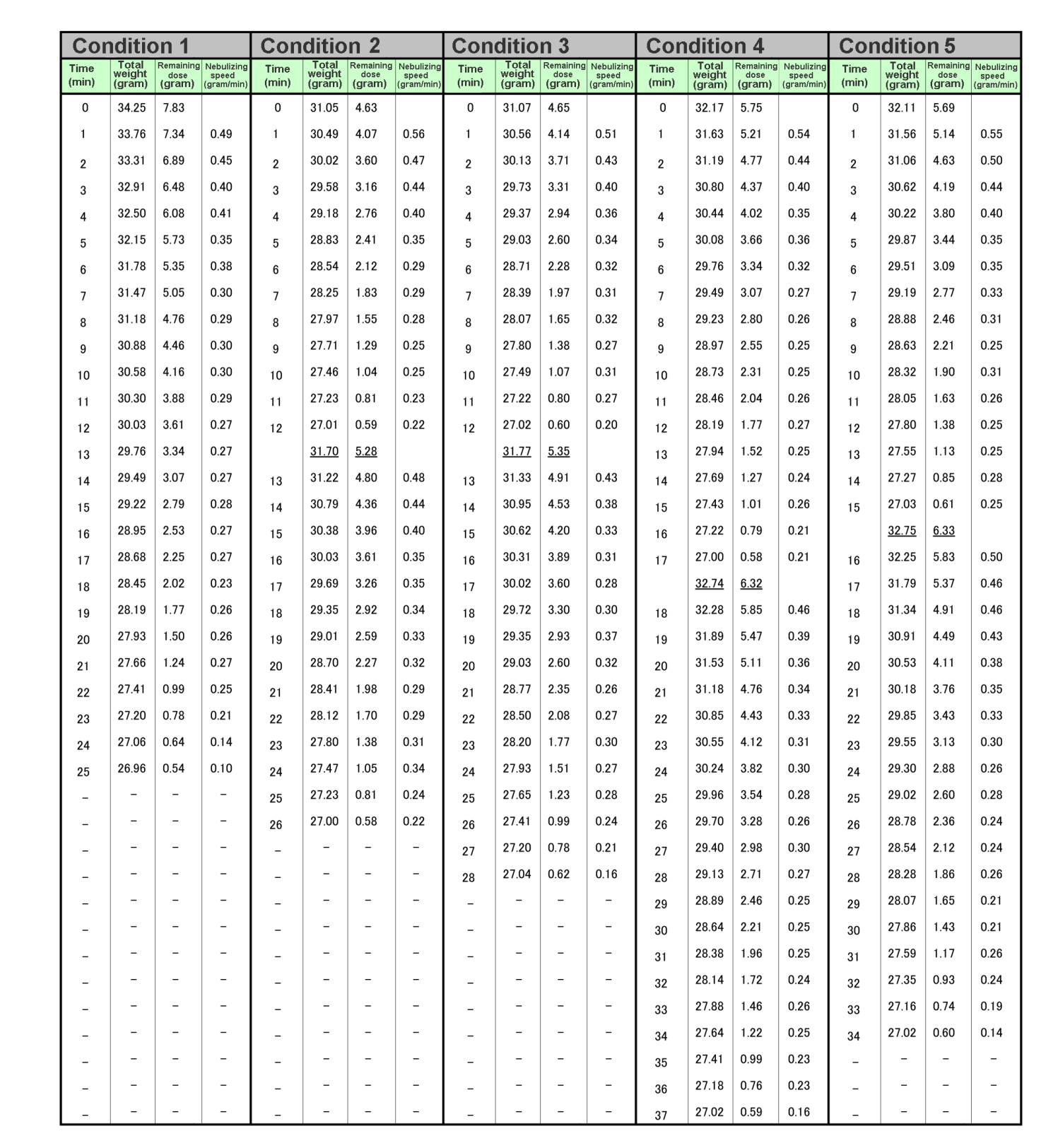

Supplement: Supplementary file 3 — Time course changes in the total weights, remaining doses and nebulizing speeds. The underlined numbers indicate the weight data measured immediately after the amikacin solution was added. (DOCX 677 kb) [file 12879_2017_2665_MOESM3_ESM.docx]
