# Supplementary material for: The efficacy, safety, and feasibility of inhaled amikacin for the treatment of difficult-to-treat non-tuberculous mycobacterial lung diseases
Source: BMC Infect Dis. 2017 Aug 9;17:558. doi: 10.1186/s12879-017-2665-5 (PMC5550988; doi:10.1186/s12879-017-2665-5)
Supplement: Supplementary file 4 — The equipment used to measure the aerosolized particle sizes (Mastersizer 2000: Malvern Instruments Ltd., Worcestershire, UK). (DOCX 251 kb) [file 12879_2017_2665_MOESM4_ESM.docx]

**
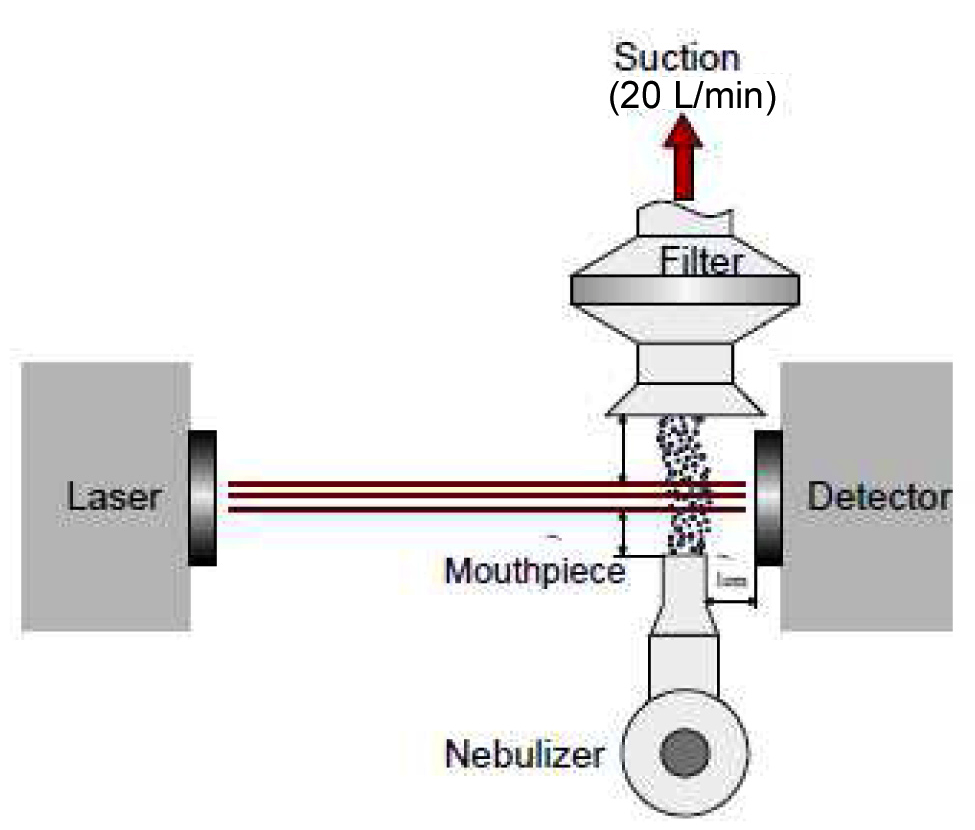
**

**Figure S1** The equipment used to measure the aerosolized particle sizes (Mastersizer 2000: Malvern Instruments Ltd., Worcestershire, UK).
