# Supplementary material for: The efficacy, safety, and feasibility of inhaled amikacin for the treatment of difficult-to-treat non-tuberculous mycobacterial lung diseases
Source: BMC Infect Dis. 2017 Aug 9;17:558. doi: 10.1186/s12879-017-2665-5 (PMC5550988; doi:10.1186/s12879-017-2665-5)
Supplement: Supplementary file 5 — Particle sizes and solution volumes in condition 1, condition 2 and condition 5. The aerosolized particle sizes were measured using a Mastersizer 2000 (Malvern Instruments Ltd., Worcestershire, UK). (DOCX 243 kb) [file 12879_2017_2665_MOESM5_ESM.docx]

**
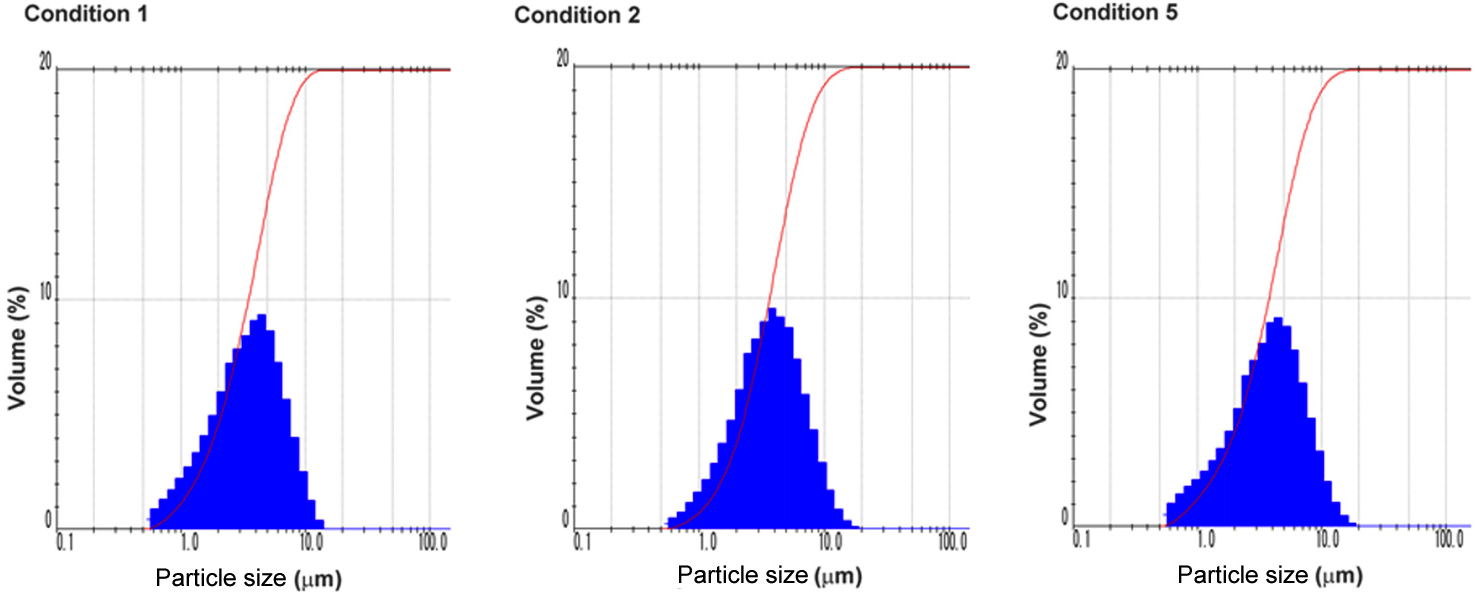
**

**Figure S2** Particle sizes and solution volumes in condition 1, condition 2 and condition 5. The aerosolized particle sizes were measured using a Mastersizer 2000 (Malvern Instruments Ltd., Worcestershire, UK).
