# Supplementary figures and images for: The efficacy, safety, and feasibility of inhaled amikacin for the treatment of difficult-to-treat non-tuberculous mycobacterial lung diseases
Source: BMC Infect Dis. 2017 Aug 9;17:558. doi: 10.1186/s12879-017-2665-5 (PMC5550988; doi:10.1186/s12879-017-2665-5)

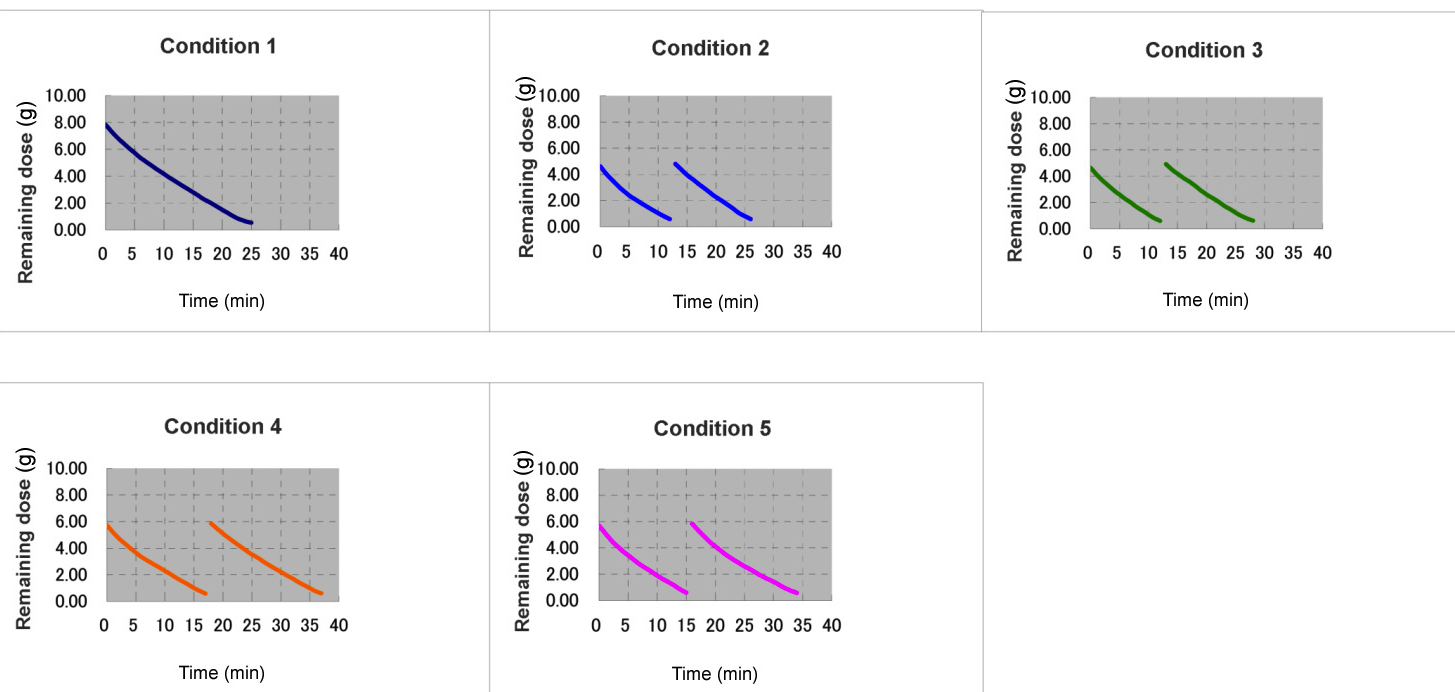


**Figure S3**  Time course changes of the remaining doses.

Supplement: Supplementary file 6 — Time course changes of the remaining doses. (DOCX 282 kb) [file 12879_2017_2665_MOESM6_ESM.docx]

**
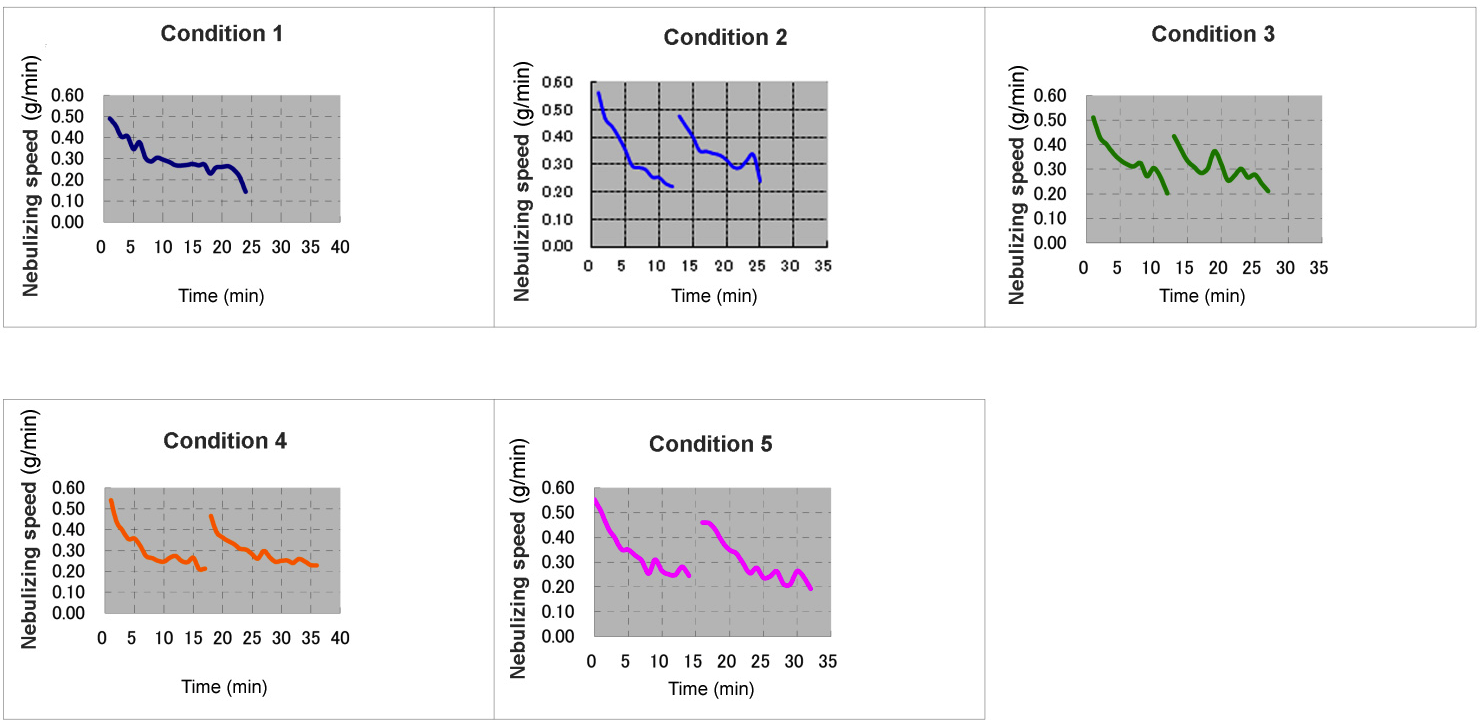
**

**Figure S4**  Time course changes of the nebulizing speeds.

Supplement: Supplementary file 7 — Time course changes of the nebulizing speeds. (DOCX 312 kb) [file 12879_2017_2665_MOESM7_ESM.docx]
